# The Effect of Ellagic Acid on Hepatic Lipid Metabolism and Antioxidant Activity in Mice

**DOI:** 10.3389/fphys.2021.751501

**Published:** 2021-10-07

**Authors:** Qiuying Xu, Shuwei Li, Wenjie Tang, Jiayou Yan, Xiaolan Wei, Mengjia Zhou, Hui Diao

**Affiliations:** ^1^Sichuan Nursing Vocational College, Chengdu, China; ^2^Animal Breeding and Genetics Key Laboratory of Sichuan Province, Sichuan Academy of Animal Science, Chengdu, China; ^3^Sichuan Animtech Biology Development Co., Ltd, Chengdu, China; ^4^Livestock and Poultry Biological Products Key Laboratory of Sichuan Province, Sichuan Animtche Feed Co. Ltd, Chengdu, China

**Keywords:** ellagic acid, lipid metabolism, antioxidant activity, liver, mice

## Abstract

Accumulating evidence has demonstrated that the imbalance of lipid metabolism and antioxidant capacity leads to damage to liver. The present study aimed to investigate the effects of ellagic acid (EA), a phenolic compound, on hepatic lipid metabolism and antioxidant activity in mice. In our study, 24 C57BL/6J mice were divided into three groups: (1) control (CON); (2) basal diet+0.1% EA (EA1); and (3) basal diet+0.3% EA (EA2). After the 14-day experiment, the liver was sampled for analysis. The results showed that 0.3% EA administration increased the liver weight. Total cholesterol and low-density lipoprotein cholesterol activities decreased and high-density lipoprotein cholesterol activity increased by EA supplementation. Meanwhile, dietary supplementation with EA dose-dependently decreased the acetyl-CoA carboxylase protein abundance and increased the phospho-hormone-sensitive lipase, carnitine palmitoyltransferase 1B, and peroxisome proliferator-activated receptor alpha protein abundances. Moreover, EA supplementation reduced the malonaldehyde concentration and increased the superoxide dismutase and catalase concentrations. The protein abundances of phospho-nuclear factor-E2-related factor 2, heme oxygenase-1, and NAD(P)H: quinone oxidoreductase 1 increased by EA supplementation in a dose-dependent manner. Taken together, EA supplementation promoted the lipid metabolism and antioxidant capacity to maintain the liver health in mice.

## Introduction

The state of equilibrium of lipids content and composition is controlled by lipid metabolic pathways in the body (Seebacher et al., 2020). Lipid metabolism is complex and involved in a variety of metabolic processes, usually including lipid transport, absorption, synthesis, oxidation, and catabolism (Fernández-Ruiz, 2020). Lipid metabolism is composed of the metabolism of fatty acids (FA), triglycerides (TG), cholesterol, and other lipids (Anand, 2020; Mutlu et al., 2021). FA metabolism is fundamental to other lipid metabolisms, which can be converted into other lipids through anabolism. Other lipids can also be converted into FA through catabolism, which will be further oxidized and decomposed into smaller FA chains through β-oxidation and generate energy (Poitelon et al., 2020). However, the metabolism of TG and cholesterol is related to the lipid transfer protein, including high-density lipoprotein cholesterol (HDL-C) and low-density lipoprotein cholesterol (LDL-C). Both HDL-C and LDL-C can combine with lipids, which were transported to different parts of the body for metabolism (Joseph et al., 2014). Maintaining normal metabolism and energy homeostasis is strictly dependent on lipid metabolism. However, the cases of dyslipidemia increased in recent decades. Dyslipidemia is part of metabolic syndrome in the body, resulting in excessive or insufficient lipids in various tissues (Bahiru et al., 2021). Intracellular dyslipidemia includes abnormal lipid regulation, accumulation of TG, and free fatty acids (FFA). Extracellular dyslipidemia includes abnormal lipid deposition and abnormal secretion of adipokines (Fu et al., 2019). Dyslipidemia can lead to obesity, type 2 diabetes mellitus, atherosclerosis (AS), and other diseases (Vekic et al., 2019; Wang et al., 2020; Valanti et al., 2021). Moreover, lipid peroxidation is a common oxidation reaction of polyunsaturated FA in living organisms. Excessive oxidation products are harmful to proteins, lipids, and nucleic acids, leading to the damage to cellular structure and function (Cháfer-Pericás, 2021). Thereby, reactive oxygen species-induced lipid peroxidation could contribute to chronic diseases, such as cardiovascular disease, diabetes, cancer, and senescence (Clemente et al., 2020; Gianazza et al., 2020; Kuzgun et al., 2020). Due to the side effects of drug treatment on dyslipidemia and lipid peroxidation, finding bioactive compounds from natural products has attracted more and more attention.

Ellagic acid (C_14_H_6_O_8_, EA), an active natural polyphenol ingredient, is extensively found in a variety of fruits and nuts (such as pomegranate, gallnut, blueberry, and grape; Zuccari et al., 2020). The molecular structure of EA contains multiple phenolic hydroxyl groups with strong antioxidant functions (Dávalos et al., 2019). In addition, a large number of studies have confirmed that EA has antibacterial, anti-inflammatory, liver protection, prevention and treatment of obesity, and anti-tumor effects (Chen et al., 2018). Furthermore, dietary supplementation with EA substantially decreased *de novo* synthesis of FA in mature adipocytes and hepatic TG levels by downregulating adipogenic markers, such as CCAAT/enhancer-binding protein alpha and peroxisome proliferator-activated receptor gamma (Okla et al., 2015; Woo et al., 2015).

As mentioned above, several investigations have expounded that EA can modulate lipid metabolism and enhance antioxidant activities. However, to our knowledge, the underlying molecular mechanisms of EA on lipid metabolism and oxidative damage in mice have not been fully elucidated. Thus, the present study aims to evaluate the possible hepatoprotective effects of EA and explore the underlying mechanisms *in vivo*.

## Materials and Methods

The experimental procedures in the study were approved by the Ethics Review Committee for Animal Experimentation of Sichuan Academy of Animal Science (Chengdu, China; P202002028). All experimental protocols were performed following the practical animal protection law and the Guide for the Care and Use of Laboratory Animals formulated by the National Research Council (China).

### Animal Care and Experimental Design

C57BL/6J mice (weighing 26~30*g*) were supplied by the Dossy Experimental Animals Co., Ltd (Chengdu, China). The mice were kept in an animal room at a constant temperature (25±2°C) and humidity (55±10%) with 12h of light per day and allowed *ad libitum* to food and water before the experiment. In a 14-day experiment, 144 mice were randomly assigned to three treatments with eight replications per treatment (six mice per replication): control (*n*=48), basal diet; EA1 (*n*=48), basal diet supplemented with 0.1% EA; and EA2 (*n*=48), basal diet supplemented with 0.3% EA in our experiment.

### Slaughter and Sample Collection

After the experiment, the mice were anesthetized by intraperitoneally injecting 500μl of pentobarbital sodium (8mg/kg BW). Following this, liver tissues were harvested for subsequent analysis.

### Biochemistry Assay

Hepatic tissues were homogenized in saline and the supernatant was collected after centrifuged at 2500*g* for 10min. The quantitation of TG, LDL-C, HDL-C, total cholesterol (T-CHO), catalase (CAT), total antioxidant capacity (T-AOC), superoxide dismutase (SOD), and malonaldehyde (MDA) was conducted by enzyme-linked immunosorbent assay (ELISA) using spectrophotometric kits following the manufacturer’s instructions (Nanjing Jiancheng Bioengineering Institute, Nanjing, China).

### Histology

Liver tissue was fixed in a 4% paraformaldehyde solution for 48h, then dehydrated and embedded in paraffin, cut into 5-μm-thick sections, and stained with hematoxylin and eosin (H&E). The liver tissues were photographed under a light microscope at optical magnifications of 100×.

### Western Blotting Analysis

Frozen liver samples (approximately 0.1*g*) were homogenized using 1ml RIPA buffer. Following this, ultrasonication was performed to break the cells. The lysates were then centrifuged at 10000rcf for 20min at 4°C. The proteins in the supernatant were diluted with 4×Laemmli sample buffer (Bio-RAD, United States) and denatured in a 98°C metal bath for 10min. Equal amounts of samples were then subjected to SDS-PAGE, and the abundances of NAD(P)H: quinone oxidoreductase 1 (NQO1), heme oxygenase-1 (HO-1), phospho-nuclear factor-E2-related factor 2 (p-Nrf2), phospho-hormone-sensitive lipase (p-HSL), carnitine palmitoyltransferase 1B (CPT1B), acetyl-CoA carboxylase (ACC), peroxisome proliferator-activated receptor alpha (*PPARα*), and β-Actin proteins were assessed by Western blot using the indicated antibodies. The expression level of β-Actin was assessed to ensure equal protein sample loading. Antibodies to NQO1, HO-1, p-Nrf2, p-HSL, ACC, *PPARα,* and β-Actin were bought from the Proteintech, Wuhan, Hubei, China. Antibody to CPT1B was bought from the Thermo Fisher Scientific, Waltham, M.A, United States.

### Statistical Analysis

SPSS 24.0 statistical software (IBM, USA) was used to analyze all the data. If the data conformed to a normal distribution and the variance was homogeneous, the LSD method was used after one-way ANOVA for multiple comparisons. Otherwise, Tamhane’s T2 multiple comparisons were adopted. The data are displayed as the means±standard deviation. Data were considered to be statistically significant when values of *p*<0.05.

## Results

### Liver Weight

**Figure 1:**
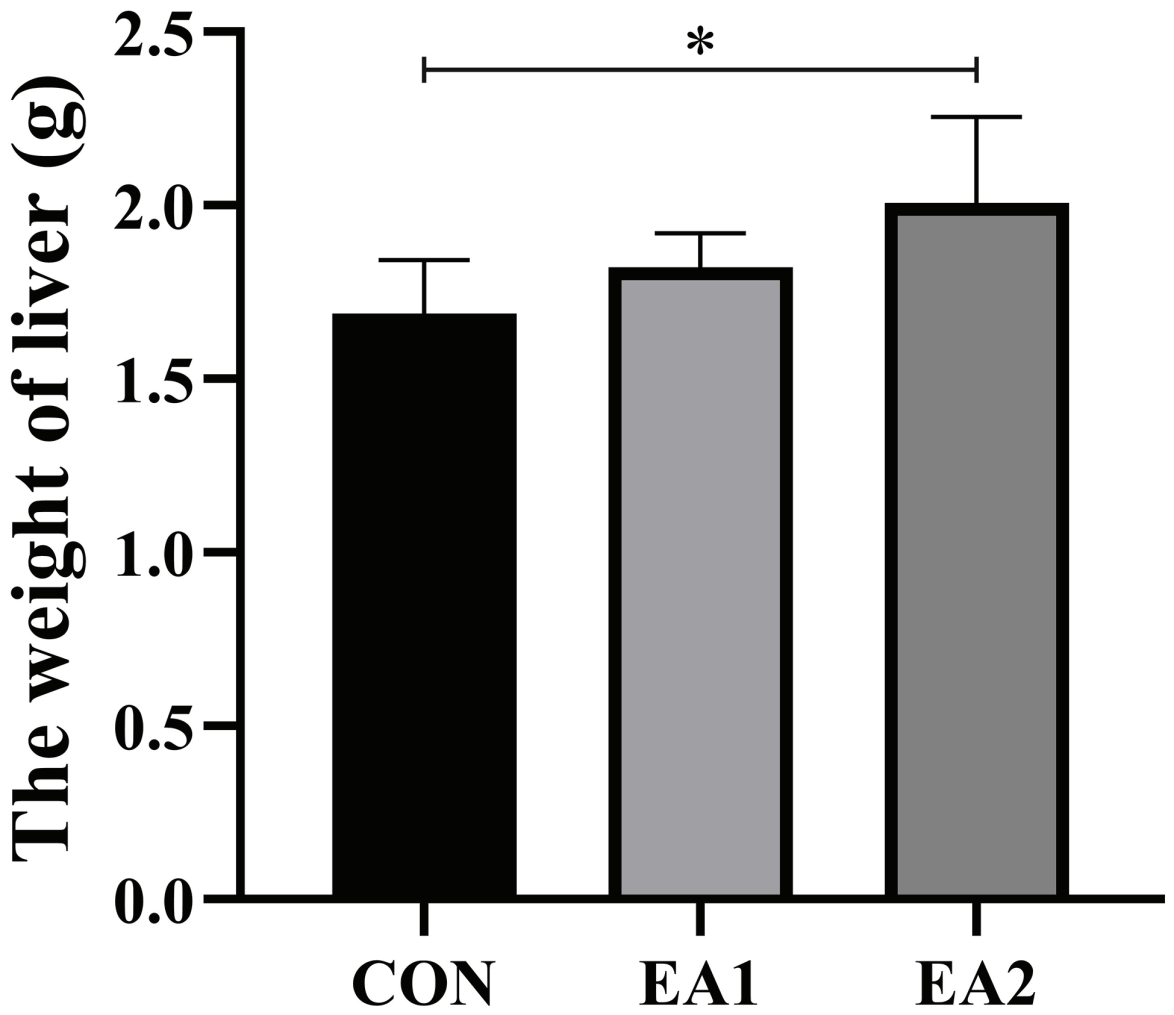
Effects of EA supplementation on liver weight in C57BL/6J mice. The values are reported as the mean±SD of 12 mice per group: ^*^p<0.05 represents significant differences compared with the control group.

The result of the effects of EA on hepatic weight was shown in Figure 1. Compared with the control group, dietary supplementation with EA increased (*p*<0.05) the weight of the liver in a dose-dependent manner.

### Hepatic Morphology

**Figure 2:**
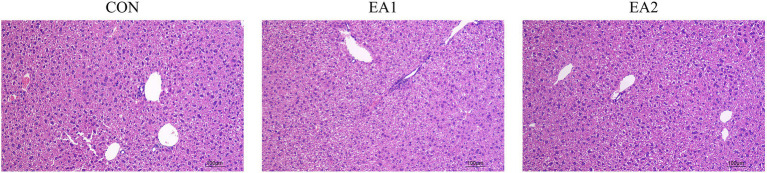
Effects of EA supplementation on the liver as disclosed by morphological analysis (H&E staining, magnifications of 100×).

The histological analysis showed that structurally ordered tissues and hepatocytes with prominent nuclei and uniform cytoplasm were observed in the control group (Figure 2A). At the same time, 0.1 and 0.3% EA treatments improved the morphological structure and integrity of normal hepatocytes (Figures 2B,C).

### Hepatic Lipid Profiles

**Figure 3:**
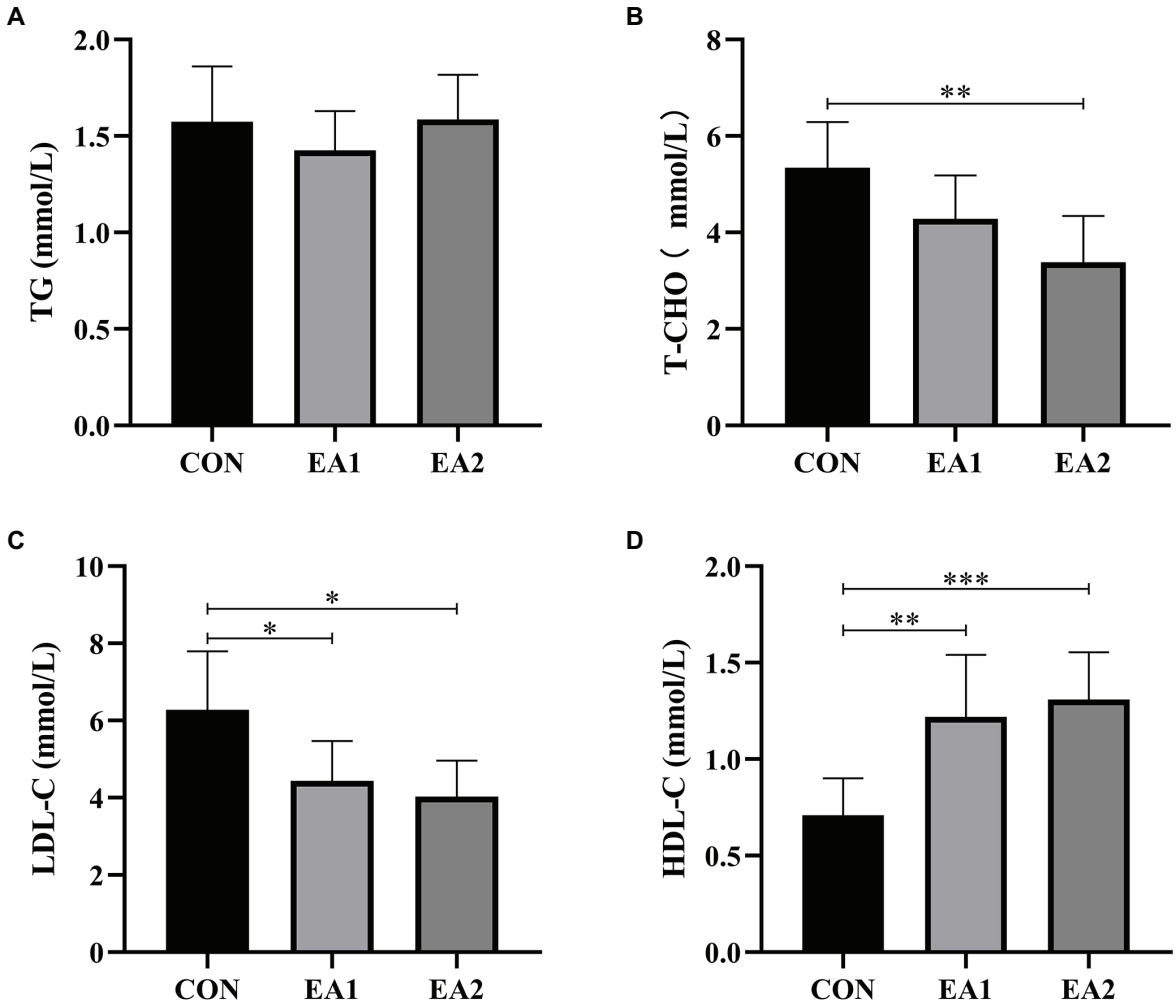
Effects of EA supplementation on lipid profiles in liver. The contents of hepatic triglyceride (TG) **(A)**, total cholesterol (T-CHO **(B)**, low-density lipoprotein cholesterol (LDL-C) **(C)**, and high-density lipoprotein cholesterol (HDL-C) **(D)** were determined by the ELISA kits. The values are reported as the mean±SD of 12 mice per group: ^*^p<0.05, ^**^p<0.01, and ^***^p<0.001 represent significant differences compared with the control group.

The results showed that dietary supplementation with 0.3% EA markedly decreased (*p*<0.01) the concentrations of T-CHO (Figure 3B) compared with the control group. However, dietary EA supplementation did not affect the concentrations of TG (Figure 3A). Moreover, dietary supplementation of EA dose-dependently decreased (*p*<0.05) the concentrations of LDL-C (Figure 3C) and increased (*p*<0.01) concentrations of HDL-C (Figure 3D).

### Lipid-Metabolism-Related Protein Abundances

**Figure 4:**
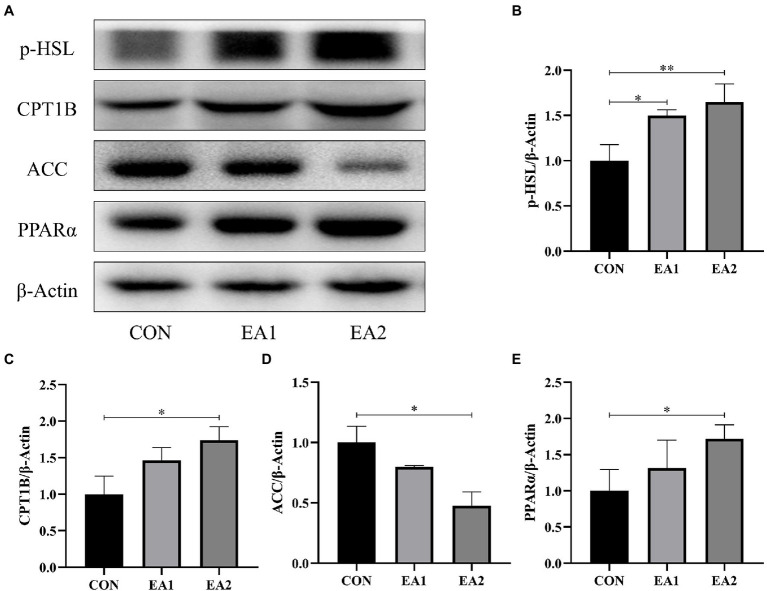
Effects of EA supplementation on lipid-metabolism-related protein abundances. **(A)**The liver samples were analyzed by Western blotting using antibodies against phospho-hormone-sensitive lipase (p-HSL), carnitine palmitoyltransferase 1B (CPT1B), acetyl-CoA carboxylase (ACC), peroxisome proliferator-activated receptor alpha (*PPARα*), and β-Actin (used as the protein loading control). **(B)** The p-HSL/β-Actin ratio in the liver. **(C)** The CPT1B/β-Actin ratio in the liver. **(D)** The ACC/β-Actin ratio in the liver. **(E)** The *PPARα*/β-Actin ratio in the liver. ^*^p<0.05 and ^**^p<0.01 represent significant differences compared with the control group.

In Figure 4, we detected the abundances of four proteins (p-HSL, CPT1B, ACC, and *PPARα*) in the process of lipid metabolism (Figure 4A). Compared with the control group, 0.1 and 0.3% EA supplementation decreased (*p*<0.01) the hepatic protein abundance of ACC (Figure 4D) and increased (*p*<0.01) the hepatic protein abundances of p-HSL (Figure 4B), CPT1B (Figure 4C), and *PPARα* (Figure 4E) in C57BL/6J mice.

### Hepatic Antioxidant Capacity

**Figure 5:**
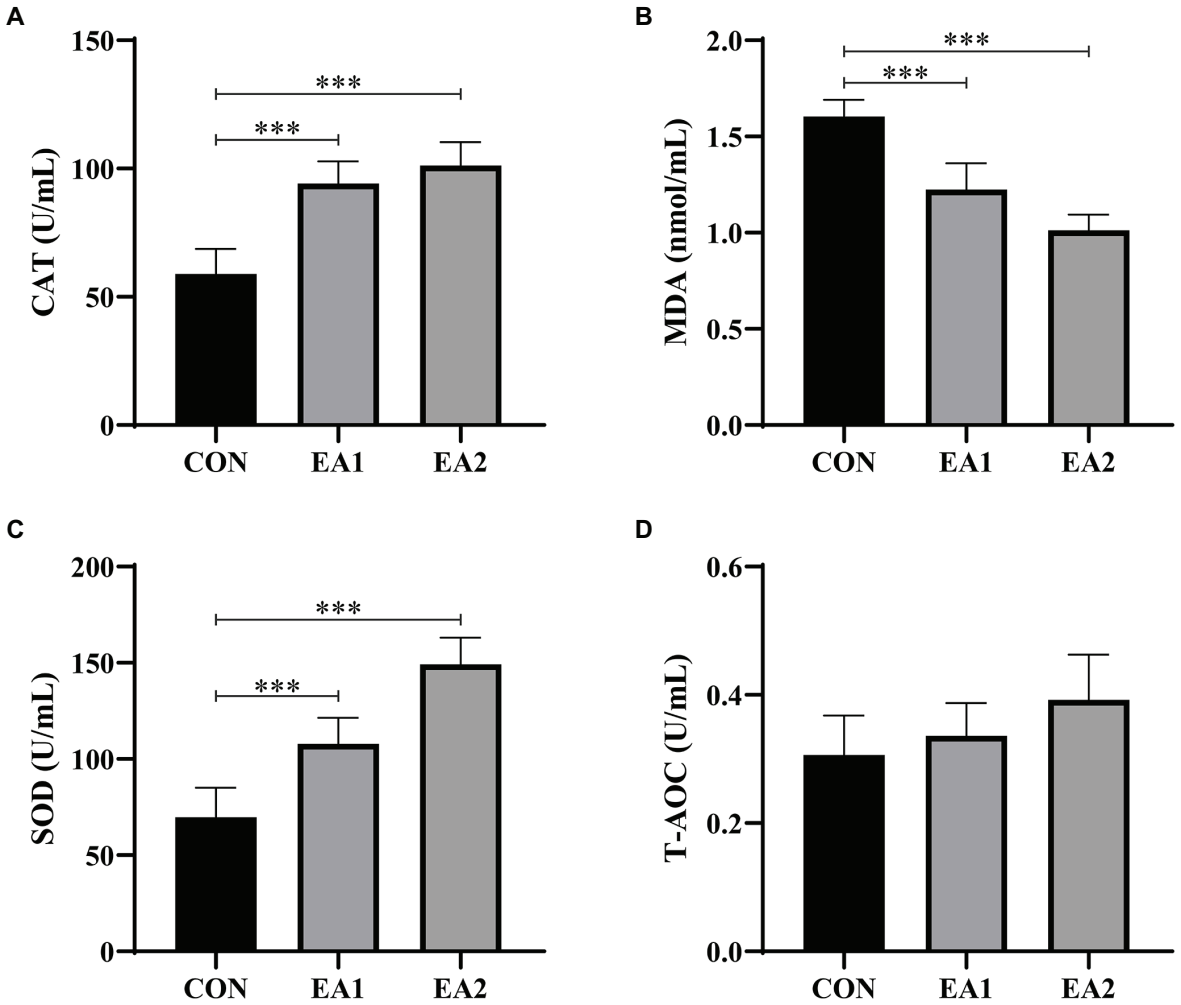
Effects of EA supplementation on antioxidant activities in liver. The contents of hepatic catalase (CAT) **(A)**, malonaldehyde (MDA) **(B)**, superoxide dismutase (SOD) **(C)**, and total antioxidant capacity (T-AOC) **(D)** were determined by the ELISA kits. The values are reported as the mean±SD of 12 mice per group: ^***^p<0.001 represents significant differences compared with the control group.

According to Figure 5, it is found that dietary supplementation with EA decreased (*p*<0.001) the concentration of MDA (Figure 5B) and increased (*p*<0.001) the concentrations of CAT (Figure 5A) and SOD (Figure 5C) in a dose-dependent manner, without (*p*>0.05) any effect on the concentration of T-AOC (Figure 5D) in comparison with the control group.

### Nrf2-ARE Signaling-Related Protein Abundances

**Figure 6:**
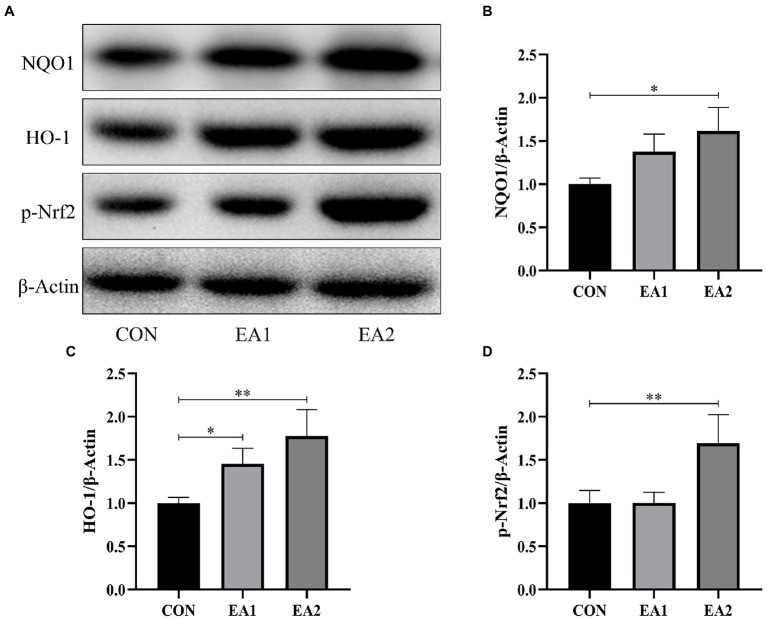
Effects of EA supplementation on Nrf2-ARE signaling-related protein abundances. **(A)** The liver samples were analyzed by Western blotting using antibodies against NAD(P)H: quinone oxidoreductase 1 (NQO1), heme oxygenase-1 (HO-1), phospho-nuclear factor-E2-related factor 2 (p-Nrf2), and β-Actin (used as the protein loading control). **(B)** The NQO1/β-Actin ratio in the liver. **(C)** The HO-1/β-Actin ratio in the liver. **(D)** The p-Nrf2/β-Actin ratio in the liver. ^*^p<0.05 and ^**^p<0.01 represent significant differences compared with the control group.

The expressions of hepatic antioxidant enzymes were strongly regulated by Nrf2-ARE Signaling. Hence, we detected the protein abundances of NQO1, HO-1, and Nrf2, which play important role in the Nrf2-ARE Signaling. From the results of Figure 6, it was found that dietary supplementation with 0.1 and 0.3% EA increased (*p*<0.01) the hepatic protein abundances of NQO1 (Figure 6B), HO-1 (Figure 6C), and p-Nrf2 (Figure 6D) in C57BL/6J mice.

## Discussion

The imbalance of fat deposition and consumption caused by the dysfunction of lipid metabolism often leads to excessive fat deposition, which is detrimental to the health of animals and humans. Dyslipidemia is often manifested as a variation of hyperlipidemia indicators, such as serum TC, TG, abnormal elevation of LDL-C, or abnormal reduction of HDL-C (Tietge, 2014). Hyperlipidemia is not only the causative factor for many cardiovascular diseases, such as atherosclerosis, coronary artery disease, and myocardial infarction but also closely related to obesity, fatty liver, hypertension, and hyperglycemia (Alloubani et al., 2020; Yang et al., 2020). Hence, maintaining the balance of lipid metabolism is vital to the health of animals and humans. Previous studies have demonstrated that EA might serve as a potential agent in the therapy of hepatic steatosis in patients with NAFLD by activation of AKT/mTORC1 signaling in the liver (Zhang et al., 2019). Our results demonstrated that dietary supplementation with EA significantly increased the concentration of HDL-C and decreased the concentrations of T-CHO and LDL-C, which, to some extent, supported the notion that EA administration ameliorated the dysfunction of lipid metabolism. The molecular mechanisms, including lipogenesis, lipolysis, and fatty acid oxidation (FAO), are complex and critical for lipid metabolism. Therefore, we further explored whether EA supplementation affects the process of lipid metabolism through these key steps in the liver of mice. ACC, a biotin-dependent allosteric carboxylase, is the rate-limiting enzyme of the process of *de novo* fatty acid synthesis and catalyzes the first reaction in the fatty acid synthesis pathway (Hunkeler et al., 2018). As a neutral lipase, HSL mainly catalyzes the hydrolysis of TG, diacylglycerol (DG), and monoacylglycerol (MAG) in animal adipose tissue, releasing FFA to participate in the body oxidation energy supply (Recazens et al., 2020). Besides, CPT1 is the rate-limiting step in the process of FA transportation into mitochondria. Overexpression of CPT1B improved insulin sensitivity and FAO to lower lipid contents (Khan and Alhomida, 2013). Meanwhile, *PPARα* can regulate the activity of CPT1B. Our data showed a dramatic increase in the hepatic protein abundances of p-HSL, CPT1B, and *PPARα* and a marked decrease in the hepatic protein abundance of ACC, indicating that EA promoted the lipid metabolism by improving hepatic lipolysis and FAO and suppressing hepatic lipogenesis. However, dietary supplementation with 0.1% EA showed little effect on the lipid metabolism comparing with 0.3% EA, which possibly supported the notion that a higher concentration of EA possessed a better function in regulating the lipid metabolism of the liver.

Previous research has demonstrated that chronic oxidative stress is considered one of the critical factors responsible for the liver injury (Rezzani and Franco, 2021). Lipid peroxidation resulting from oxidative stress produces a large amount of MDA, which can also contribute to hepatocyte damage, thereby aggravating the lesions (Tsikas, 2017). By contrast, SOD and CAT are important antioxidant enzymes, responsible for scavenging free radicals and inhibiting lipid peroxidation, thus protecting the hepatocytes against toxicity (Mansuroğlu et al., 2015; Nowicki and Kashian, 2018). A kinetic DFT study found that EA was more reactive toward HO•, but less reactive toward CCl_3_OO• with good rate constants for the reactions (Tošović and Bren, 2020). Moreover, EA could restore the antioxidant defense system by scavenging free radicals, leading to the abatement of oxidative damage in the livers and brains of D-gal-induced aging (Chen et al., 2018). Therefore, the antioxidant capacity of EA was assessed in this study. Dietary supplementation with EA dose-dependently increased the contents of CAT and SOD and decreased the content of MDA, implying EA exerts a protection effect by elevating the hepatic antioxidant capacity. It is reported that the elevation of antioxidant activity was regulated by several critical antioxidant genes. Nrf2 is the most critical transcription factor for intracellular ROS clearance and the most important defense system against oxidative stress caused by pathogen infection (Tian et al., 2020). Nrf2 regulates the expression of the antioxidant gene mainly by nuclear translocation and interacting with the antioxidant response element (ARE) at the 5′ end of the gene (Johnson et al., 2008). After the ARE is activated, a series of antioxidant proteins/enzymes, such as NQO1 and HO-1, are induced to maintain the redox balance (Liang et al., 2013). Our study suggested that dietary supplementation with EA increased the hepatic protein abundances of p-Nrf2, NQO1, and HO-1. These results implied that dietary EA supplementation could enhance the antioxidant capacity by elevating the Nrf2-ARE signaling pathway.

## Conclusion

To summarize, our study demonstrated that dietary supplementation with 0.1 and 0.3% EA has beneficial effects on lipid metabolism by regulating the proteins expressions related to lipogenesis, lipolysis, and FAO. Besides, the enhanced antioxidant capacity caused by EA is relevant to the activation of the Nrf2-ARE signaling pathway. Our results provided a scientific basis for the potential contribution of EA to maintain liver health.

## Data Availability Statement

The original contributions presented in the study are included in the article/supplementary material, and further inquiries can be directed to the corresponding author.

## Ethics Statement

The animal study was reviewed and approved by the Ethics Review Committee for Animal Experimentation of Sichuan Academy of Animal Science (Chengdu, China).

## Author Contributions

HD: conceptualization, methodology, validation, resources, writing—review and editing, supervision, and funding acquisition. QX: formal analysis, investigation, and writing—original draft preparation. SL, WT, and JY: data curation. XW and MZ: project administration. All authors have read and agreed to the published version of the manuscript.

## Funding

This work was supported by the Sichuan Science and Technology Program (2021NZZJ0019).

## Conflict of Interest

Author SL was employed by company Sichuan Animtech Biology Development Co., Ltd, and authors WT, JY, XW, and MZ were employed by company Sichuan Animtche Feed Co. Ltd.The remaining authors declare that the research was conducted in the absence of any commercial or financial relationships that could be construed as a potential conflict of interest.

## Publisher’s Note

All claims expressed in this article are solely those of the authors and do not necessarily represent those of their affiliated organizations, or those of the publisher, the editors and the reviewers. Any product that may be evaluated in this article, or claim that may be made by its manufacturer, is not guaranteed or endorsed by the publisher.
